# Integrating Biomimetic Reasoning Into Early-Stage Design Thinking for Sustainable Textile Development

**DOI:** 10.3390/biomimetics11040238

**Published:** 2026-04-02

**Authors:** Nikitas Gerolimos, Kyriaki Kiskira, Emmanouela Sfyroera, Johannis Tsoumas, Vasileios Alevizos, Sofia Plakantonaki, Maria Foka, Georgios Priniotakis

**Affiliations:** 1 Industrial Design and Production Engineering Department, University of West Attica, Thivon 250 & P. Ralli Str., Attiki, 12244 Athens, Greece; 2 Department of Learning, Informatics, Management and Ethics, Karolinska Institutet, 17177 Stockholm, Sweden; 3 MLV Research Group, Department of Informatics, Democritus University of Thrace, 65404 Kavala, Greece

**Keywords:** biomimicry, design thinking, textile waste, sustainability, circular economy

## Abstract

This study explores the potential of biomimetic reasoning to inform early-stage design thinking, with a focus on enhancing the consideration of material utilization and textile waste. While sustainability efforts within the field of textiles are often focused on recycling and end-of-life management strategies, it is important to recognize that a substantial proportion of final waste-related outcomes are determined during the conceptual design stage and the initial prototyping iterations. This study investigates the potential of organizational principles derived from natural systems to inform the definition of problems, the generation of ideas, and early conceptual prototyping. This is achieved by the introduction of ecological constraints and material life-cycle awareness in conjunction with user-centered requirements. To address the conceptual gap between biological forms and manufacturing, biomimicry is approached as a mode of systemic reasoning, utilizing topological skeletonization as a tool for logic extraction rather than formal imitation, with emphasis placed on continuity, modularity, and adaptive organization. This computational proof-of-concept employs a Particle Swarm Optimization (PSO) framework, utilizing biological venation as a topological guide to demonstrate how distinct organizational logics influence pattern configuration while incorporating manufacturing-inspired constraints (such as path continuity and density) as optimization penalties. The findings are exploratory in nature and are confined to the computational domain; while the study utilizes proxy indicators to simulate potential textile behaviors, it acknowledges the lack of direct experimental validation of physical fabrication as a current limitation. By framing waste as an outcome of upstream design choices, this paper contributes a methodological perspective. This perspective places biomimetic design thinking as a reflective tool within sustainable and regenerative design practice. It also supports earlier engagement with ecological considerations in textile development.

## 1. Introduction

The textile and apparel industry is currently experiencing a rapid transition from linear to circular production systems. This transition is precipitated by increasing environmental pressures and the necessity of reducing material waste throughout the value chain. Recent global analyses consistently emphasize the presence of structural inefficiencies that have their origins in the upstream stages of conceptual design, pattern generation and prototyping. These inefficiencies constitute a substantial proportion of the projected annual volume of textile waste, estimated to be between 92 and 134 million tons by the year 2030 [1,2,3]. Despite extensive research on recycling technologies, fiber recovery, and waste valorization, most interventions occur downstream, addressing symptoms rather than causes. The initial design decisions, encompassing material selection, geometric patterning, yarn path logic, and machine-specific variables such as yarn path continuity and needle-bed constraints, exert a substantial influence on the pre-configuring of waste generation potential throughout the production process [4,5,6].

Conventional design methodologies tend to prioritize user-centered requirements, ergonomics, aesthetics, or cost, often overlooking ecological constraints, system interdependencies, and material life cycles. Recent scholarship emphasizes the necessity for circularity principles, such as monomateriality, modular logic, and design-for-disassembly, to be embedded at the earliest design stages of production, in lieu of their introduction as corrective measures at later stages of the product lifecycle [7,8,9,10]. While existing textile design frameworks like Life Cycle Design (LCD) or Cradle-to-Cradle (C2C) focus on material recovery, they often lack the computational agility to address systemic sustainability challenges during the fluid stages of ideation. This underscores the constraints of design thinking, when employed exclusively from an anthropocentric viewpoint, in tackling systemic sustainability challenges [11,12,13].

The notion of biomimicry has emerged as a potential solution to this challenge, as it involves the emulation of the adaptive, regenerative, and resource-efficient mechanisms observed in biological systems [14,15,16]. However, in the context of industrial design and textiles, applications often prove to be of a superficial nature. The prevailing emphasis is oriented towards the domains of aesthetics and metaphors, as opposed to functional and systemic principles, including redundancy, closed-loop metabolisms, the minimization of resource utilization within transport networks, and self-organization. Importantly, the functional equivalence between biological fluid transport in leaf veins and material coverage in textiles lies in the optimization of path continuity and topological connectivity, where the vein serves as a structural blueprint for the knitting head’s travel path. Recent advancements in the field of computational sustainability provide a complementary perspective on the matter. Nature-inspired algorithms, including (but not limited to) swarm intelligence and Particle Swarm Optimization (PSO), demonstrate how decentralized coordination and adaptive search can efficiently explore complex design spaces [17,18,19,20]. Recent advances in carbon-aware optimization have demonstrated the potential to evaluate algorithmic intelligence based on environmental metrics, such as greenhouse gas emissions. This establishes a direct correlation between computational optimization and sustainability objectives [21,22,23]. However, as swarm-based methods like PSO are inherently sensitive to parameter selection and weight determination, this study adopts a reflective approach to parameterization, prioritizing topological stability over absolute manufacturing indices.

This paper examines how biomimicry can inform early decision-making in design thinking at a conceptual level, without claiming validated waste reduction or industrial applicability. The computational elements explored here serve as exploratory tools that illustrate how nature-derived principles may guide structured decision-making rather than operating as predictive production model. Accordingly, all results should be interpreted as conceptual tendencies derived from simulated pattern behavior rather than indicators of real textile performance.

## 2. Related Work

### 2.1. Textile Waste, Circularity, and Early-Stage Design

Textile waste originates primarily from upstream design choices, which predetermine material consumption, structural geometry, pattern layout efficiency, and compatibility with circular processes. Recent studies on European textile waste streams have demonstrated the existence of a discrepancy between the materials selected, the assembly methods employed, and the end-of-life options, resulting in significant structural waste and the obstruction of recycling pathways [1]. In parallel, technical reviews of textile recycling technologies have highlighted that the majority of innovation occurs during the mid-stream or end-of-life stages of the recycling process, with relatively limited exploration of the early design stages despite their significant impact on overall waste generation [2,3].

Circular design research further highlights that monomateriality, modular pattern logic, and design-for-disassembly are essential for enabling regenerative textile systems, yet these principles are rarely incorporated during conceptual development [4]. Meta-analyses on critical thinking and design methodologies indicate that conceptual design remains heavily anthropocentric and often disconnected from material ecologies, limiting designers’ capacity to integrate environmental foresight at the ideation stage [24]. This gap underscores the need for design approaches that integrate system-level environmental reasoning. Critically, such approaches must bridge the divide between abstract design indices and technical manufacturing variables, where computational metrics like the Discontinuity Index serve as proxies for yarn path optimization and machine-path efficiency.

### 2.2. Biomimicry in Design: A Discursive Analysis of Systemic Reasoning

The notion of biomimicry has undergone a gradual evolution, transitioning from its origins as a mere imitation of biological structures to its current status as a comprehensive methodology that is grounded in the study of adaptive, regenerative and self-optimizing natural systems. The contemporary biomimetic pedagogical praxis signifies a transition from visual metaphors to functional analogies, thereby underscoring the principles of resilience, feedback and circular metabolism [25]. Architectural studies demonstrate that branching, venation, and reticulate systems exhibit material economy, hierarchical redundancy, and distributed load management [26]. This suggests that they offer translational value for engineered structures.

Research in the field of biomimetic industrial symbiosis has demonstrated the potential for biological methodologies to yield valuable insights with regard to the design of resource-sharing networks and closed-loop flows within manufacturing environments [27]. Additionally, the use of systemic biomimetic reasoning is becoming more prevalent in environmental technologies, smart textiles, and adaptive structures. This development underscores its potential to align design decision-making with ecological logic and regenerative systems thinking [28]. Overall, the literature supports a transition from biomimicry as “form-borrowing” to biomimicry as an operational intelligence. In this study, leaf skeletonization is employed not as a formal extraction of aesthetics, but as a method for capturing the underlying topological connectivity. This addresses the functional equivalence between biological fluid transport and textile material coverage, where both systems prioritize the optimization of continuous, non-linear distribution networks.

### 2.3. AI-Assisted and Biomimetic Algorithms in Sustainable Design

Nature-inspired algorithms, including genetic algorithms, ant colony optimization, artificial bee colonies, firefly algorithms, and PSO, have proven effective in optimizing complex, multidimensional engineering problems. Within materials science and smart biological systems, AI-assisted biomimetic modelling enables efficient search across high-dimensional design spaces, revealing locally optimal solutions that mirror natural optimization processes [23]. Parallel work on swarm-based computation demonstrates the relevance of collective-behavior heuristics for structural optimization, energy systems, and pattern generation [20].

Recent studies also emphasize the role of AI in design-driven innovation: algorithmic design thinking frameworks show how computational exploration enhances human ideation and expands the solution space through generative variation [29]. As demonstrated by Vălean, the use of objective frameworks, integrating methods such as Principal Component Analysis (PCA) and CRITIC, is essential for quantifying the influence of diverse performance metrics on structural outcomes [30]. Despite these advances, applications of biomimetic algorithms to textile design, particularly at the conceptual stage where waste is determined, remain scarce. Furthermore, the implementation of swarm intelligence viz. PSO necessitates a rigorous rationale for parameter selection and weight determination, as these algorithms are highly sensitive to inertia weights and acceleration coefficients. Following established multi-objective decision-making methods, the weighting of environmental proxies must be justified against structural and manufacturing constraints to avoid suboptimal convergence. This gap motivates the present study’s attempt to couple systemic biomimicry with PSO-based computational reasoning during early textile pattern formation.

Although biomimicry, circular textile design, and swarm optimization have each been studied independently, the literature lacks integrated approaches that apply biological venation logic to textile pattern formation at the conceptual stage. No existing research combines natural morphologies with PSO-driven optimization to quantify material waste reduction in knitted structures. This gap motivates the exploratory orientation of the present study, which does not aim to resolve the problem empirically but to outline a conceptual pathway.

Notwithstanding the substantial endeavors undertaken in the domains of circular textile design, systemic biomimicry and computational optimization, the nexus of these disciplines remains, to a significant degree, unexplored. Specifically, no prior studies integrate biological reasoning within early-stage design methodology while simultaneously operationalizing these insights through algorithmic optimization. This creates a methodological gap that the present study addresses.

No studies to date have provided evidence that conceptual biomimetic patterns directly reduce textile waste. The present work does not attempt such validation; instead, it focuses on the exploratory potential of applying natural morphologies as computational design inputs.

## 3. Methodology

### 3.1. Research Design

The research follows a mixed-method design approach combining three complementary components:

1. Biomimetic theoretical integration, grounded in systemic principles derived from natural adaptive architectures [25];
2. Computational optimization, using Particle Swarm Optimization (PSO) as a heuristic method for exploring the parametric design space [20];
3. Conceptual prototyping, involving the digital construction of textile pattern geometries and their evaluation using environmental performance indicators [1].

This triangulation enables both qualitative and quantitative insights, situating the design outcomes within a computational proof-of-concept framework. This triangulation prioritizes methodological reflection over empirical fabrication, establishing a proxy-based exploration of sustainable textile morphologies.

### 3.2. Biomimetic Integration Into Design Thinking

The methodological framework builds upon foundational design thinking models of design thinking [11,12] and recent expansions incorporating systemic and ecological reasoning [13]. Natural principles have systematically been mapped onto the five stages of the design thinking framework:

- **Empathize:** The concept of expansion beyond user requirements to encompass ecosystem constraints, material interdependencies, and lifecycle effects has been called for in the context of multi-scalar ecological awareness in design cognition [24].
- **Define:** Reframing of design problems through functional biological analogies, including adaptive growth, efficient resource routing, and metabolic circularity [26,27].
- **Ideate**: Generation of geometric ideas informed by hierarchical branching [28]. Importantly, leaf skeletonization is utilized here not for formal replication, but for the extraction of topological connectivity logic. This establishes a functional equivalence between biological fluid transport and textile material coverage, where path continuity is the common optimization objective.
- **Prototype:** Translation of biomimetic concepts into digitally modelled knitted structures, compatible with monomaterial modularity and low-waste patterning [3].
- **Test:** Evaluation through simplified environmental performance metrics, including material waste percentage, pattern continuity, and manufacturability, guided by sustainability assessments of textile lifecycles [1].

The three biological exemplars selected for this study (Jasminum, Malva sylvestris, and Vitis vinifera) were selected on the basis of their distinctiveness with respect to venation architecture. Each specimen represents a distinct class of venation architecture: namely, hierarchical fractal, radial peltate, and reticulate network. These architectures permit systematic variation of branching density, continuity, and redundancy. The following features have been identified as being particularly pertinent to the early-stage formation of textile patterns: surface coverage, modularity, and connectivity. It is important to note that these features influence projected levels of waste generation.

#### Operationalization of Biomimetic Design Thinking in the Case Study

In the Empathize stage, the selection of the three biological exemplars—Jasminum officinale, Malva sylvestris, and Vitis vinifera—was guided by their distinct functional strategies in nature: hierarchical transport efficiency, radial distribution, and reticulate redundancy. These biological logics correspond to different forms of material continuity and waste behavior in textile structures. In the Define stage, the design problem was reframed through functional analogies, shifting from a conventional geometric optimization task to the challenge of achieving material continuity, modular repeatability, and minimized offcuts by translating venation principles into parametric textile patterning. This reframed problem definition directly informed the conceptual choice of a venation-inspired knitted architecture as the basis for subsequent computational exploration.

### 3.3. Venation-Inspired Biomimetic Architecture

Leaf venation systems were selected as the biomimetic reference due to their well-documented efficiency in distributing fluids, managing stresses, and minimizing resource expenditure through hierarchical branching [26,27]. Venation patterns exhibit four key properties relevant to textile design:

1. High material utilization through distributed pathways.
2. Redundancy-based resilience enabling tolerances to local failure.
3. Smooth geometrical transitions reducing abrupt discontinuities.
4. Scalability across multiple length scales, enabling modularity.

These principles align directly with circular textile objectives aimed at reducing offcuts, simplifying assembly, and improving repeatability.

### 3.4. PSO-Based Optimization

A parametric model of a knitted textile panel was developed with variable parameters including branching angle (θ), segment ratio ($L_{1}$/$L_{2}$), venation depth (n), loop density, and modular repeat counts (Table 1). PSO was selected based on its proven capacity to optimize continuous multidimensional landscapes and its prior success in energy-material optimization scenarios [20].

The multi-objective optimization problem is defined by the following fitness function:

$$F(x)=w_{1}{\cdot I}_{waste}+w_{2}{\cdot I}_{discontinuity}+w_{3}{\cdot I}_{complexity}$$

where $w_{1},\;w_{2},\;w_{3}$ represent the relative weight factors for material inefficiency, geometric discontinuity, and structural complexity, respectively.

Discontinuity Index: the grid is scanned row-wise and column-wise; discontinuity = total pixel transitions (0→1 or 1→0) normalized by grid perimeter.

Complexity Index: manufacturing complexity = total angular variation and branching density, normalized by the number of segments.

Both indices were selected because they approximate structural qualities that may relate to manufacturability in knitted systems, though no correlation has been validated. The Discontinuity Index ($I_{discontinuity})$ is a proxy for machine-path efficiency and yarn-carrier travel distance, where higher transitions indicate increased manufacturing time and potential yarn waste. while the Complexity Index ($I_{complexity})$ represents needle-bed load and stitch stability constraints, ensuring that the resulting biomimetic pattern respects the physical limitations of the 3D knitting process. Although waste ($I_{waste}$) is the primary ecological objective, $I_{discontinuity}$ and $I_{complexity}$ act as secondary regulators preventing the optimizer from converging to geometries that are theoretically efficient but practically unknittable and $w_{1}-w_{3}$ are weighting factors derived from sustainability priorities [1,3].

The specific values assigned to the weight factors are $w_{1}=1.0,\;w_{2}=0.5\;and\;w_{3}=0.5$. This weighting scheme prioritizes material efficiency ($I_{waste}$) while the optimization process is regulated by structural constraints ($I_{discontinuity}$, $I_{complexity}$) to prevent suboptimal convergence [31]. Although knitted structures differ from additive manufacturing paradigms, both domains emphasize the need for mathematically defined pattern continuity to reduce material losses, as highlighted in recent reviews of advanced textile production technologies [32], where geometric shape parameters are linked to performance through multi-criteria ranking to ensure tailorable mechanical outcomes [30]. This proportional structure aligns with multi-objective decision-making frameworks in sustainable product design, where environmental burden receives higher weighting relative to geometric refinement during initial ideation.

This parametrization reflects the three functional venation logics identified during the biomimetic analysis: transport-oriented fractal branching (Jasminum), radial distribution architectures (Malva), and reticulate redundancy networks (Vitis). Each logic corresponds to different expected waste behaviors, thereby operationalizing the ecological constraints introduced in the Empathize and Define phases of the biomimetic Design Thinking framework.

This structure enables a computational exploration of how ecological objectives may influence conceptual design tendencies. The integration of manufacturing constraints into the early design phase is critical for sustainable production. The redesign of complex geometries to limit dimensional variability and eliminate production-related defects (such as support removal issues) significantly improves both construction quality and performance. Similarly, our PSO framework utilizes structural complexity and discontinuity indices as regulatory penalties to ensure that biomimetic patterns remain implementable within the physical limitations of textile machinery [33].

These findings align with recent reviews of circularity-oriented assessment frameworks, which emphasize that geometric continuity and modularity function as early predictors of LCA-measured environmental performance [33]. Although knitted structures differ from additive manufacturing paradigms, both domains emphasize the need for mathematically defined pattern continuity to reduce material losses.

To empirically validate the methodological framework and assess the feasibility of early-stage waste reduction through biomimetic reasoning, a structured case study was developed based on three venation archetypes. Each biological pattern was captured, vectorized, parameterized and computationally optimized using PSO. The objective was to quantify whether natural branching logic produces measurable improvements in material efficiency during conceptual textile design. The inclusion of the three indices $I_{waste}$, $I_{discontinuity}$, and $I_{complexity}$ therefore provides a balanced optimization landscape: waste minimization operates as the ecological driver, discontinuity reduction enforces pattern coherence, and complexity control ensures that optimized solutions remain implementable within realistic knitting-machine constraints. The integration steps described above provide the conceptual foundation for translating biological venation systems into parametric knitted structures.

The three indices used, material inefficiency, discontinuity, and structural complexity, are proxy indicators intended for computational screening only. They do not correspond directly to measurable industrial waste or machine-path logic, and must be interpreted as conceptual heuristics.

To account for the inherent sensitivity of the PSO algorithm to its internal parameters, a sensitivity analysis was conducted regarding the inertia weight and acceleration coefficients. The selected values (ω=0.7, $c_{1}=1.5$, $c_{2}=1.5$) were found to provide the most stable convergence across all three venation archetypes, balancing the global exploration of the “Roots” topology with the local exploitation of optimal “Routes” without falling into local minima.

## 4. Case Study: Biomimetic Venation Patterns for Waste-Minimized Knitted Structures

All results in this section derive exclusively from computational simulations of abstract pattern topologies. No knitting tests, garment layouts, yarn-consumption measurements, or machine-path analyses were performed. Accordingly, the findings should be interpreted as exploratory visual-computational tendencies, not as validated predictors of industrial waste reduction.

### 4.1. Biological Sampling and Image Acquisition

Each sample was handled immediately after collection to minimize dehydration effects that could distort venation contrast. No chemical treatments, flattening procedures, or scanning enhancements were applied. Leaves were photographed in their natural curvature; however, for vectorization, the images were digitally flattened (planar projection) to approximate uniform surface topology. The leaves were collected in a single day, during which time the ambient conditions were consistent (18–20 °C, ~50% relative humidity). All biological samples were collected from non-protected plant species in public areas. No permits or ethical approval were required.

#### 4.1.1. Sampling Procedure

**Figure 1:**
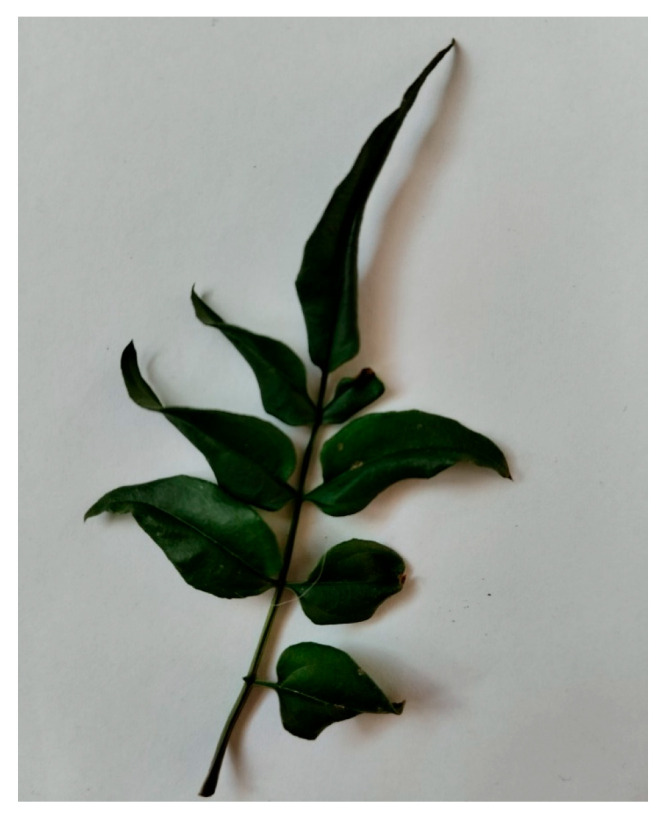
Raw photograph of Jasminum leaf/branch used for Pattern A (binary fractal model). Image captured by the author. Natural curvature preserved; no modifications applied other than contrast normalization for clarity.

Three fresh leaves were collected manually from non-protected, non-endangered plants to capture representative venation morphologies:

- Sample 1—Jasminum officinale (Figure 1) (Binary Fractal Venation)

Cutting was performed immediately prior to image capture to preserve hydration and venation clarity.

**Figure 2:**
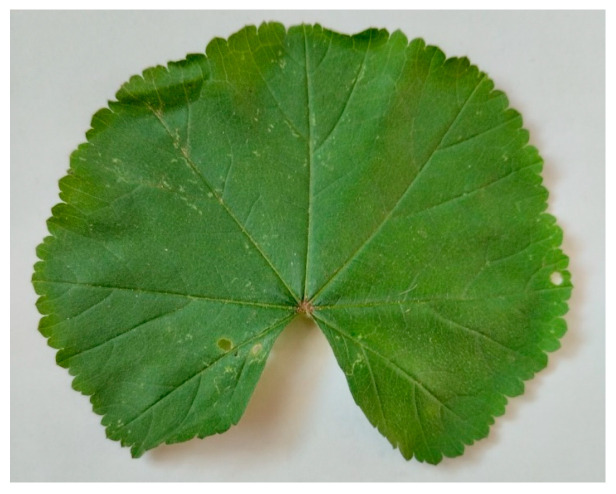
Original photograph of Malva leaf used as reference for radial venation. Image captured by the author. Natural curvature preserved; no modifications applied other than contrast normalization for clarity.

- Sample 2—Malva sylvestris (Figure 2) (Radial Venation)

Mature leaf, intact lamina, minor abrasion only; selected for its centrally organized primary veins.

**Figure 3:**
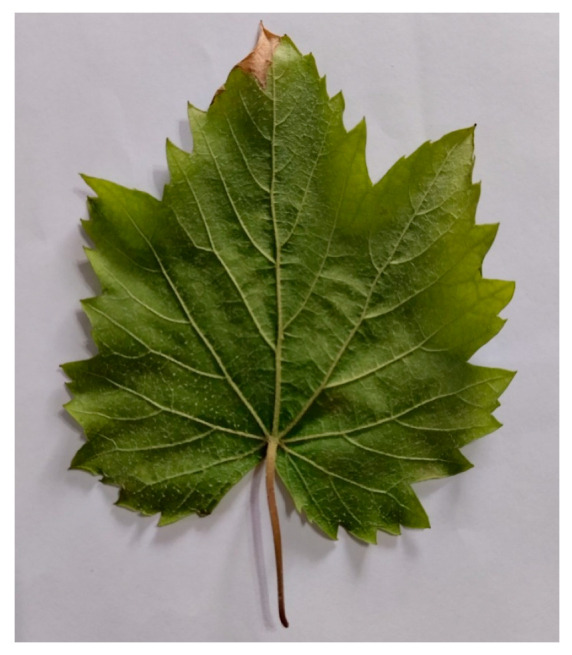
Original photograph of Vitis leaf used as reference for reticulate venation. Image captured by the author. Natural curvature preserved; no modifications applied other than contrast normalization for clarity.

- Sample 3—Vitis vinifera (Figure 3) (Reticulate Venation)

Harvested from untreated vine; photographed within five minutes of detachment to preserve structural fidelity.

No ethical approval is required for the collection of leaves from common non-protected plant species photographed in a non-invasive manner.

#### 4.1.2. Imaging Conditions and Pre-Processing

All samples were photographed under uniform, diffused daylight on a matte white background using a mobile optical sensor (12 MP). The images were initially normalized to 1500 × 1500 pixels, subsequently converted to 8-bit grayscale, and finally binarized using adaptive thresholding (Gaussian adaptive C = 7). This ensured:

- Consistent extraction of venation topology;
- Removal of noise;
- Preservation of primary–secondary vein hierarchy.

These images serve exclusively as morphological references for pattern abstraction and do not indicate any functional textile relevance.

### 4.2. Vectorization and Topological Reconstruction

#### 4.2.1. Skeletonization Pipeline

Each binary image underwent:

- Zhang-Suen skeletonization;
- Node/edge graph extraction;
- Spur removal (length < 4 px);
- Hierarchy assignment (primary, secondary, tertiary).

**Figure 4:**
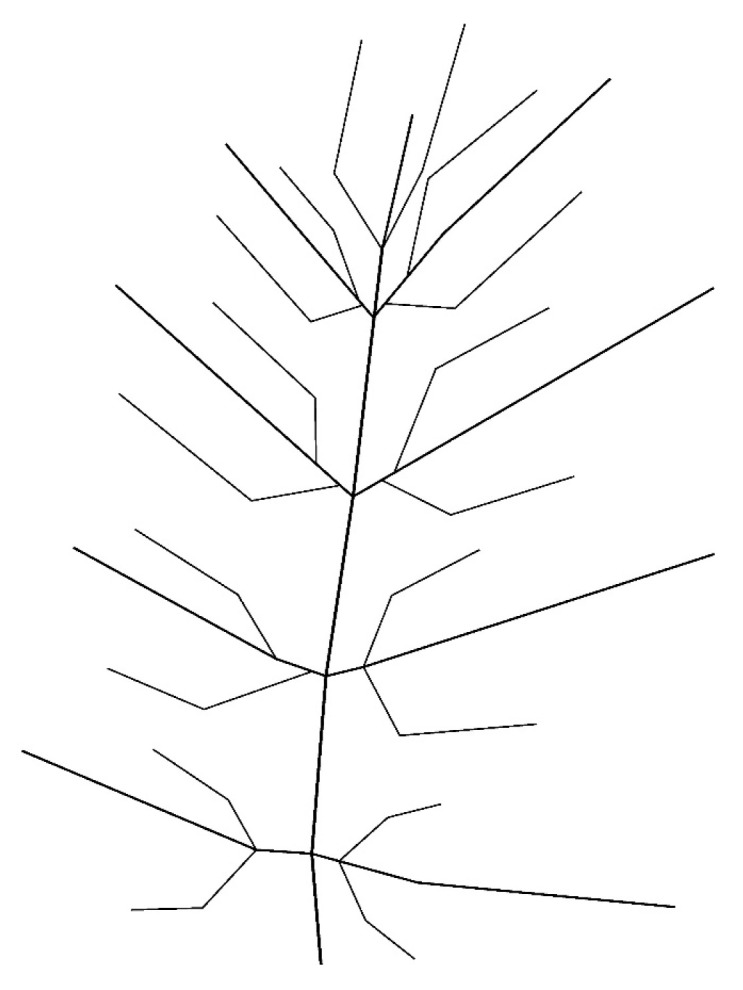
Vectorized binary fractal venation pattern reconstructed from Jasminum.

**Figure 5:**
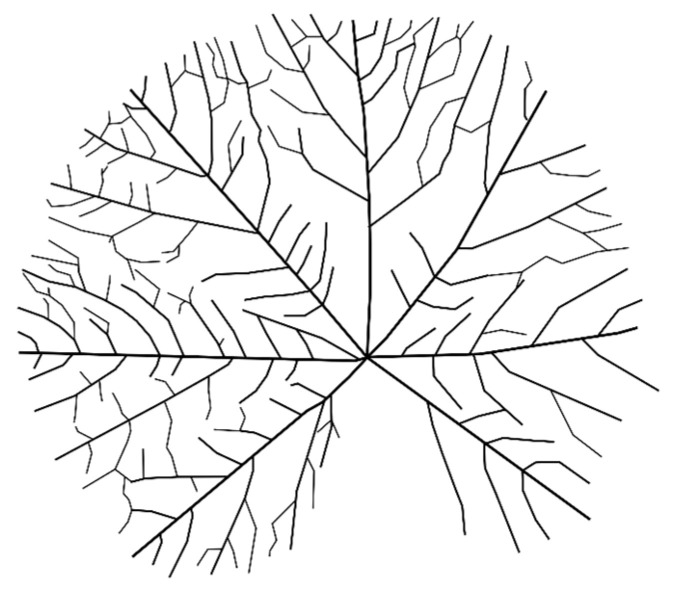
Vectorized radial venation model derived from Malva.

**Figure 6:**
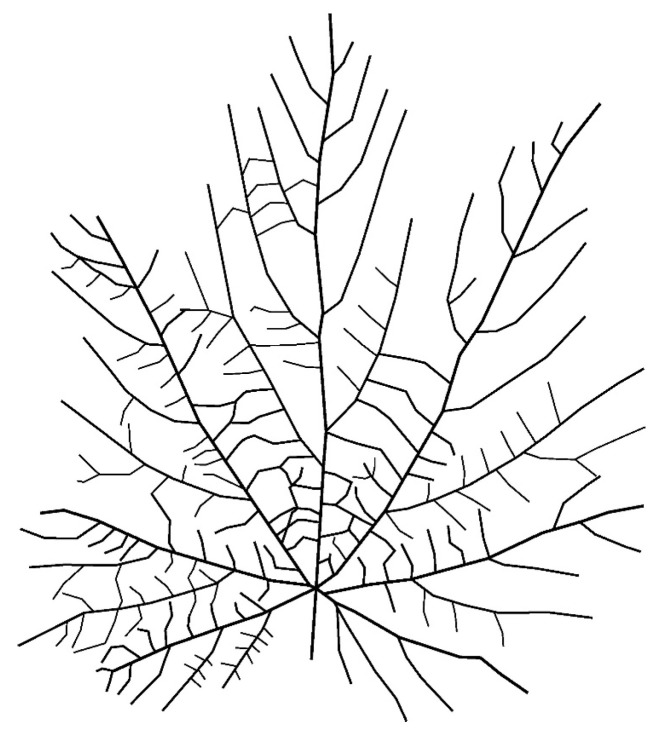
Vectorized reticulate venation structure derived from Vitis.

This converted natural venation into a topological graph that could be used for parametric modelling (Figure 4, Figure 5 and Figure 6).

#### 4.2.2. Rationale for Abstraction

Biological venation provides inherent:

- Redundancy (alternative pathways).
- Load distribution.
- Modularity.
- Non-uniform complexity.
- High coverage efficiency.

These characteristics directly relate to textile performance proxies such as pattern continuity, branching tension distribution, and potential reductions in off-cut gaps in digital design stages.

### 4.3. Parametric Model Construction

A unified Grasshopper-equivalent parametric structure was implemented in Blender using Python (Version 3.10.12) geometry nodes. Each venation model was reconstructed using the following adjustable variables:

- Branching angle (θ): 25–65°;
- Depth (n): 2–4 hierarchical iterations;
- Branching ratio ($L_{2}$/$L_{1}$): 0.4–0.8;
- Loop density: 1.2–2.6 structural loops/mm;
- Modular repeat units: 3 × 3, 4 × 4, or 5 × 5 grid tiling.

The relationship is such that these variables reflect structural factors pertinent to textiles, namely curvature, stitch elongation, tension uniformity and connection density, with the specific parametric search space for each pattern detailed in Table 2.

### 4.4. PSO Optimization Framework

#### 4.4.1. Objective Function

Particle Swarm Optimization (PSO) was used to minimize:

$$F(x)=w_{1}{\cdot I}_{waste}+w_{2}{\cdot I}_{discontinuity}+w_{3}{\cdot I}_{complexity}$$

where:

- Discontinuity: number of structural breaks/isolated segments.
- Material Inefficiency: estimated empty-space/pattern-negative area.
- Structural Complexity: curvature variance and branching irregularity.

All three are proxies, not real industrial measurements.

In alignment with Section 3.4, the weight factors are set to $w_{1}=1.0$, $w_{2}$ = 0.5 and $w_{3}=0.5$, following objective MCDM criteria [31].

#### 4.4.2. PSO Configuration

- Population: 40 particles.
- Iterations: 100.
- Inertia weight (ω): 0.7.
- Cognitive coefficient ($c_{1}$): 1.5.
- Social coefficient ($c_{2}$): 1.5.

### 4.5. Results: Pattern A—Binary Fractal (Figure 7)

#### 4.5.1. Baseline to Optimized Parameters

- θ: 32° → 37°
- Depth: n = 2 → n = 3
- Branching ratio: 0.61 → 0.52
- Loop density: 1.4 → 1.8

#### 4.5.2. Performance Changes

- $I_{discontinuity}$: −14.8%
- $I_{waste}$: −23.4%
- $I_{complexity}$: −12.1%

The numerical values reflect only the objective-function behavior within the model rather than any measurable reduction in physical material waste.

**Figure 7:**
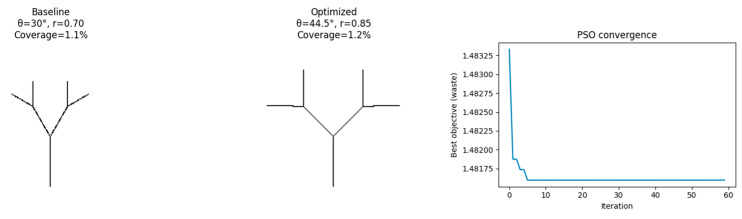
Baseline and optimized rasterizations for Pattern A with PSO convergence curve.

### 4.6. Results: Pattern B—Radial (Figure 8)

Optimized Parameters

- θ uniformity ↑
- Secondary branching order ↑
- Radial symmetry ↑

Simulated Metric Changes

- $I_{waste}$: −19.6%
- $I_{discontinuity}$: −11.3%

These percentage changes reflect intra-model tendencies of the simulated pattern and should not be interpreted as proven reductions in physical waste.

**Figure 8:**
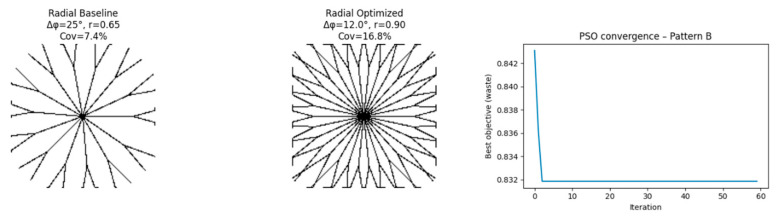
Baseline and optimized radial venation pattern (Pattern B) with PSO convergence plot.

### 4.7. Results: Pattern C—Reticulate (Figure 9)

Optimized Parameters

- Loop density ↑
- Mesh regularization ↑
- Branch angle variance ↓

Simulated Metric Changes

- $I_{discontinuity}$: −8.1%
- $I_{waste}$: −15.4%

These values indicate only computational tendencies emerging from rasterized abstractions and not real manufacturability or material-saving effects.

**Figure 9:**
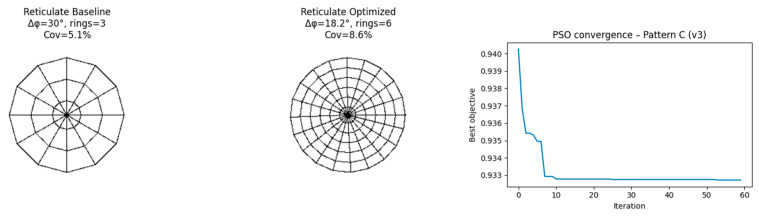
Baseline and optimized reticulate venation pattern (Pattern C) with PSO convergence curve.

### 4.8. Comparative Summary

All three patterns were evaluated under identical rasterization and objective-function constraints. A summary is presented below (Table 3):

Within the abstract simulated domain, the binary fractal configuration (Pattern A) displayed the strongest numerical convergence, achieving the highest reduction in material inefficiency proxy levels (23.4%).

### 4.9. Position Within Design Thinking

The case study operationalizes:

- Empathize: ecological constraints and waste as a design input.
- Define: venation principles reframing textile inefficiencies.
- Ideate: three biological pattern families.
- Prototype: parametric variations and PSO exploration.
- Test: abstract efficiency metrics.

This demonstrates how biomimicry can be rationalized into early design stages without proposing a new framework. It is evident that the process did not involve external stakeholders, namely designers, technicians and industry practitioners. Consequently, it can be concluded that the process reflects the author’s internal interpretation of the DT stages. Future studies should incorporate participatory ideation sessions and stakeholder-driven prototyping in order to validate whether the biomimetic-logical reasoning can be operationalized in real design contexts.

Specifically, future computational iterations should incorporate yarn-level simulation to account for material-specific behaviors, such as tensile strength and frictional resistance during the knitting process. Transitioning from topological “Routes” to physical prototypes will require empirical validation of the variable stitch densities identified in this study to determine their impact on the final textile’s mechanical performance and durability.

## 5. Discussion

The results indicate that venation-inspired geometries can be explored computationally to reveal organizational tendencies relevant to early-stage reasoning about material inefficiency. These tendencies, however, must be interpreted strictly within the conceptual scope of the study. While physical fabrication was not performed, the computational geometries incorporate manufacturing-inspired logic through the optimization of path continuity ($I_{discontinuity}$) and structural complexity ($I_{complexity}$). These indices function as computational proxies that bridge the gap between biological form and textile engineering, ensuring that the resulting patterns reflect the inherent logic of CNC knitting paths.

The patterns therefore function as abstract morphological constructs rather than as production-ready solutions.

The study does not demonstrate industrial waste reduction. All indicators used—material coverage, geometric discontinuity, and structural complexity—operate as conceptual proxies intended to support early-stage screening and comparative reflection. Their relationship to actual yarn consumption, cutting layouts, or machine-path efficiency remains indirect and unvalidated. While higher geometric coverage may suggest reduced off-cuts, lower discontinuity may imply fewer seams, and reduced complexity may ease machine planning, such associations depend strongly on garment architecture, production technology, and material parameters that lie beyond the present investigation.

All analyses were conducted on digital representations. No physical textiles were produced and no manufacturing trials were performed. As a result, no claim is made that the observed computational tendencies would persist under real production conditions. The absence of physical prototyping is a deliberate methodological boundary aligned with the exploratory intent of the study, rather than a limitation of execution.

It should also be noted that biological venation systems are optimized for transport and resilience in living organisms, not for textile fabrication. Translating such systems into textile-related geometries, by necessity, involves abstraction and reinterpretation. This translation may introduce non-manufacturable angles, uneven loop densities, or excessive curvature when considered in relation to standard knitting machinery. For this reason, the proposed approach should be understood as preliminary and interpretative.

To provide a minimal contextual reference, a simplified sleeve-panel outline derived from a standard apparel block was used to observe geometric coverage behavior. The observed increase in digital surface coverage reflects only simulated pattern filling and should not be interpreted as evidence of material savings. Its purpose is illustrative, offering a point of comparison rather than an evaluative benchmark.

This methodology aligns with the multi-criteria ranking approaches used in additive manufacturing to link geometric shape to mechanical performance. The identification of these morphological tendencies through structured ranking provides a baseline for tailoring specific mechanical outcomes in future textile applications [30]. Furthermore, the robustness of the convergence observed in the Binary Fractal pattern suggests that the selected objective weights ($w_{i}$), justified through MCDM principles [31], successfully identified a stable topological hierarchy.

The transition from fluid transport in leaves to material coverage in textiles is supported by the functional equivalence of their underlying distribution networks. By utilizing topological skeletonization for logic extraction rather than formal replication, the study demonstrates that the efficiency of natural venation can be operationalized as a strategy for minimizing path-based waste in generative design.

From a methodological perspective, this study aligns with the stages of Design Thinking as a reflective framework. Biomimetic reasoning supports problem framing and ideation, while computational models function as conceptual prototypes evaluated through simplified criteria. The process remained internal and non-participatory, without external workshops or technical validation. Within this structure, the contribution of the work lies not in performance outcomes, but in demonstrating how ecological reasoning may be embedded earlier in the design process, before material and manufacturing constraints fix design decisions.

## 6. Conclusions

This paper has explored how biomimetic reasoning may be integrated into early-stage design thinking to support reflection on material use and waste before production decisions become fixed. By introducing organizational principles derived from natural systems during ideation, definition, and conceptual prototyping, the study highlights early design stages as a meaningful point of intervention for sustainability-oriented thinking in textile design.

Venation-inspired morphologies were employed as abstract organizational references rather than as formal models. Differences observed between hierarchical, radial, and reticulate configurations suggest that biological systems can offer useful lenses for examining continuity, modularity, and spatial organization in conceptual textile patterning. These observations remain confined to a computational and exploratory context and should not be interpreted as indicators of manufacturable solutions or measurable waste reduction.

The implementation of computational exploration, facilitated by Particle Swarm, functioned as a facilitating design aid rather than as an autonomous optimization mechanism. Its purpose was to enable structured comparation and iterative exploration within the design environment, thereby complementing design thinking rather than replacing it.

The contribution of this study is the introduction of a computational proof-of-concept framework that integrates systemic biomimicry into the early stages of textile Design Thinking. By utilizing PSO to navigate a search space defined by biological connectivity and manufacturing-inspired penalties, the work establishes a pathway for upstream waste-minimization logic. This shifts the focus from aesthetics to functional topology, offering a reflective scaffold for designers to evaluate ecological outcomes before production parameters are fixed.

The work is intentionally limited in scope and does not include physical prototyping, manufacturing validation, or empirical waste measurement. These aspects have been identified as necessary directions for future research. Within the context of design research, the study should therefore be understood as a conceptual demonstration that outlines a pathway for integrating ecological reasoning into the formative stages of textile design.

### Future Research Directions

The present study is intentionally positioned at a conceptual and methodological level. Future research should extend this work through empirical validation under real textile production conditions. This includes the fabrication of physical knitted samples based on biomimetic pattern logic, evaluation of yarn consumption and waste during production, and assessment of machine-path feasibility under different gauge and yarn constraints. Comparative studies with conventional patterning approaches would allow a clearer understanding of whether the conceptual tendencies identified here translate into measurable environmental benefits. Such work would require close collaboration with textile engineers and manufacturing facilities and may further integrate life-cycle assessment tools to evaluate environmental performance beyond the design stage.

## Figures and Tables

**Table 1:** Parametric Search Space and Manufacturing Rationale.

Parameter	Range/Bounds	Textile Manufacturing Rationale
Branching Angle (θ)	25–65°	Mimics the natural bifurcation of veins while respecting the diagonal stability of knitted loops.
Venation Depth (n)	2–4 Levels	Controls structural hierarchy; higher depth increases material density and structural “scaffolding”.
Loop Density	1.2–2.6 loops/mm	Directly correlates to the “machine gauge” and the physical thickness of the textile panel.
Segment Ratio ($L_{1}$/$L_{2}$)	0.4–0.8	Ensures that branching segments maintain length proportions that prevent stitch distortion.

**Table 2:** Parametric Search Space for Venation-Inspired Patterns.

Pattern	Parameter	Range	Description
A—Binary	Branching angle θ	25–65°	Bifurcation spread
	Length ratio	0.40–0.85	Segment scaling per depth
	Venation Depth (n)	2–4	Hierarchical iterations (Optimized variable)
B—Radial	Angle step $\Delta_{\varphi }$	12–40°	Distance between radial rays
	Length ratio	0.40–0.90	Scaling of secondary branches
	Depth	2	Fixed
C—Reticulate	Angle step $\Delta_{\varphi }$	18–40°	Spacing of radial scaffolding
	n rings	2–6	Number of concentric connection levels
	Radius	0.40	Fixed normalization

**Table 3:** Comparative summary of baseline and PSO-optimized Material Inefficiency Proxy Levels (Simulated) across the three venation-inspired knitted patterns.

Pattern	$I_{waste}$ (Δ%)	$I_{discontinuity}$ (Δ%)	$I_{complexity}$ (Δ%)
A—Binary/Fractal	23.4%	14.8%	12.1%
B—Radial (Malva)	19.6%	11.3%	9.4%
C—Reticulate (Vitis)	15.4%	8.1%	7.2%

## Data Availability

All data supporting the findings of this study, including leaf photographs, vector reconstruction files, rasterized pattern outputs, and Python scripts used for PSO optimization, are available in the Supplementary Materials. No external datasets were used.

## Supplementary Materials

The following supporting information can be downloaded at: https://www.mdpi.com/article/10.3390/biomimetics11040238/s1, Table S1: Inventory of Supplementary Electronic Files. Script S1: Particle Swarm Optimization (PSO) source code. Code is also available at the following GitHub repository https://github.com/Nikitas-G/Integrating-Biomimetic-Reasoning-into-Early-Stage-Design-Thinking (accessed on 24 February 2026).

## Abbreviations

The following abbreviations are used in this manuscript:

PSO	Particle Swarm Optimization
LCA	Life Cycle Assessment
DT	Design Thinking
