# Supplementary material for: Integrating Biomimetic Reasoning Into Early-Stage Design Thinking for Sustainable Textile Development
Source: Biomimetics (Basel). 2026 Apr 2;11(4):238. doi: 10.3390/biomimetics11040238 (PMC13112943; doi:10.3390/biomimetics11040238)
Supplement: Supplementary file 1 [file biomimetics-11-00238-s001.zip › biomimetics-4200090-supplementary.pdf]

Article

Integrating Biomimetic Reasoning Into Early-Stage Design Thinking for Sustainable Textile Development

Nikitas Gerolimos [1]*, Kyriaki Kiskira [1], Emmanouela Sfyroera [1], Johannis Tsoumas [1], Vasileios Alevizos [2,3], Sofia Plakantonaki [1], Maria Foka [1] and Georgios Priniotakis [1]

Supplementary Materials

Technical Metadata for Venation Reconstructions (refer to Figures 1-6 in the main manuscript):

- Photographs: Standardized lighting, 1 cm scale reference, and orientation markers.
- Vectorization: Inkscape v1.3 using manual Bézier curve tracing (tolerance 1.5 px, node simplification 5% error tolerance).

PSO Configuration and Reproducibility (refer to Figures 7-9 in the main manuscript):

- Image processing: Python 3.10, OpenCV 4.9, scikit-image 0.22.
- Skeletonization: Zhang–Suen algorithm.
- PSO Parameters: Custom Python script (Pyswarms 1.3.0) with Population = 40, Iterations = 100, and Random seed = 42.

Data Availability: A detailed numerical summary of the parameter bounds and constraints used for Pattern A, B, and C is available at: https://github.com/Nikitas-G/Integrating-Biomimetic-Reasoning-into-Early-Stage-Design-Thinking.git

Limitations of Supplementary Data: The supplementary material documents the digital workflow and conceptual abstractions only. It does not include physical prototyping, yarn path simulations, or material properties

Table S1. Inventory of Supplementary Electronic Files

Code	File Name	Description	Format	Resolution / Metadata
Script S1	Script S1 Particle Swarm Optimization (PSO) source code	Custom Python code for the optimization of venation patterns (A, B, and C)	.py	Documentation included
